# Initial Validation of a Comprehensive Assessment Instrument for Bereavement-Related Grief Symptoms and Risk of Complications: The Indicator of Bereavement Adaptation—Cruse Scotland (IBACS)

**DOI:** 10.1371/journal.pone.0164005

**Published:** 2016-10-14

**Authors:** Catherine Newsom, Henk Schut, Margaret S. Stroebe, Stewart Wilson, John Birrell

**Affiliations:** 1 Department of Clinical Psychology, Utrecht University, Utrecht, The Netherlands; 2 Department of Clinical Psychology and Experimental Psychopathology, University of Groningen, Groningen, The Netherlands; 3 Cruse Bereavement Care Scotland, Perth, Scotland; IRCCS Istituto Auxologico Italiano, ITALY

## Abstract

**Objective:**

This study assessed the validity of the Indicator of Bereavement Adaptation Cruse Scotland (IBACS). Designed for use in clinical and non-clinical settings, the IBACS measures severity of grief symptoms and risk of developing complications.

**Method:**

*N* = 196 (44 male, 152 female) help-seeking, bereaved Scottish adults participated at two timepoints: T1 (baseline) and T2 (after 18 months). Four validated assessment instruments were administered: CORE-R, ICG-R, IES-R, SCL-90-R. Discriminative ability was assessed using ROC curve analysis. Concurrent validity was tested through correlation analysis at T1. Predictive validity was assessed using correlation analyses and ROC curve analysis. Optimal IBACS cutoff values were obtained by calculating a maximal Youden index *J* in ROC curve analysis. Clinical implications were compared across instruments.

**Results:**

ROC curve analysis results (AUC = .84, p < .01, 95% CI between .77 and .90) indicated the IBACS is a good diagnostic instrument for assessing complicated grief. Positive correlations (p < .01, 2-tailed) with all four instruments at T1 demonstrated the IBACS' concurrent validity, strongest with complicated grief measures (*r* = .82). Predictive validity was shown to be fair in T2 ROC curve analysis results (*n* = 67, AUC = .78, 95% CI between .65 and .92; p < .01). Predictive validity was also supported by stable positive correlations between IBACS and other instruments at T2. Clinical indications were found not to differ across instruments.

**Conclusions:**

The IBACS offers effective grief symptom and risk assessment for use by non-clinicians. Indications are sufficient to support intake assessment for a stepped model of bereavement intervention.

## Introduction

Researchers and practitioners alike frequently express the need for a systematic, scientifically based way to distinguish between those bereaved people who need and would benefit from intervention and others who could be expected to adjust to their loss without such aid [1]. Researchers [1, 2] estimate that some 7% of bereaved adults meet the criteria for complicated grief (CG), with dramatically higher estimates for certain risk groups. These complications can result in heightened psychological and physical distress, higher risk of mortality, suicidal ideation, and ruminative thought patterns, among other problems [3–8]. Symptoms that have been recognized as grief specific when presented by a bereaved person include yearning for the deceased person, intrusive thoughts about the deceased person and/or avoidance of reminders of him or her, anger, guilt, and the loss of a sense of meaning or purpose in life [8]. Within the first months post bereavement, it is considered normal to experience a wide range of symptom levels, from low to high [9]. Continued high symptom levels after an initial time period, estimates of which range from 2 to 6 months, have been considered indicative of complications [9,10]. In addition, specific risk factors have been recognized as increasing the likelihood that a bereaved person will develop complications in grieving. These factors include experiences such as multiple bereavements, previous trauma, or a problematic relationship with the deceased person; personal traits, such as an insecure attachment pattern; or additional current stressors, such as caregiving responsibilities, lack of social support, health problems, or substance abuse [1,11].

People with an increased risk of experiencing complications in grieving may benefit from intervention such as grief counseling and grief therapy [12,13]. In contrast, people who do not experience such complications will probably cope with their grief successfully on their own and will likely not require bereavement intervention. Identifying at-risk bereaved people seems to be both an efficient and effective strategy for bereavement support provision. To make this approach practical in an applied bereavement care context, a sound assessment process is required to help those working in the sector recognize when intervention is likely to be effective for a client.

There are good reasons to argue that, in order to be effective, a grief screening process should assess risk factors for the development of complications in grieving, as well as grief symptoms [13]. The inclusion of both grief symptoms and risk assessment in the bereavement support intake process is of particular importance in differentiated care models to ensure that commensurate levels of care are offered both to people who currently present high grief symptom levels, and those who present lower initial levels of grief symptoms but are at risk for developing high symptom levels. In addition to providing a more complete view of the complexity of a bereaved person’s case, assessing risk factors creates an opportunity for the presentation of other underlying or coexisting conditions (such as a history of abuse), which might indicate a specific focus within treatment, or, depending on the condition, a different kind of intervention altogether.

Over the previous decades, a number of well-designed grief assessment instruments have been developed and validated both for the general bereaved population and for specific subcategories of bereaved people. These include the Adult Attitudes to Grief Scale (AAGS), [14]; the Brief Grief Questionnaire (BGQ, [15]), the Inventory of Complicated Grief—Revised (ICG; [16]), the Grief Reaction Checklist [17,18], and the Texas Revised Inventory of Grief [19]. For a review of specific instruments, see [20, 21]. These instruments—all self-reports—are often used for research purposes as stand-alone assessments. In clinical practice, however, they are more likely to be used as part of a larger assessment process, including a clinical interview. The interviewer would supplement the grief symptom assessment with a professional assessment. Such an assessment would typically cover additional risk factors that might create further obstacles to coping with grief.

At present, the clinical interview remains a key component in this process. There have been no assessment indicators available to gather both grief symptom and risk information consistently outside the context of a clinical interview. Considering the limited availability of bereavement intervention in applied healthcare settings—which usually lack dedicated resources—it is often not possible for a trained clinical interviewer with knowledge of risk factors and grief symptoms to conduct intake assessments [1]. In the UK, for example, an estimated 80% to 90% of all bereavement support is provided by the voluntary (nonprofit) sector (see [22], based on the London Bereavement Network’s 2001 report on UK Standards for Bereavement Care; [23]). It has been observed that a systematic approach is lacking in the way that care is offered, including what kind of care is offered to whom, and when [24,25]. There is a demonstrated need for a comprehensive intake assessment instrument that can distinguish between clinical, subclinical, and normal levels of grief symptoms and indicate the degree of risk of complications. Such an instrument would need to be easy to use and appropriate for use by both paraprofessionals and clinicians. It would enable bereavement support organizations to provide each client with a degree of care commensurate with case complexity.

The present study offers a validation of the Indicator of Bereavement Adaptation-Cruse Scotland (IBACS). Recently developed in close consultation with professional and volunteer bereavement support practitioners, the IBACS was designed for delivery by professional practitioners as well as nonprofessional practice staff or volunteers once a concise training process has been completed. The present study focused on the IBACS’s discriminative ability and concurrent validity with existing assessment instruments, examining important aspects of the instrument’s psychometric quality (both reliability and validity). We also aimed to establish cutoff scores indicating a complicated response to grief.

Hypotheses were developed taking into consideration the IBACS’s grief-specific focus, its practitioner-informed development, and the influence of two validated, grief-specific instruments on its formation. Particular attention was given to the IBACS’s correlation with subscales assessing anxiety, depression, hyperarousal, and intrusive thoughts, all of which have been associated with grief (see [18,26–27]). In contrast, given the indications in recent literature of a weak correlation between avoidance and CG measures [28,29], lower expectations were placed around the IBACS’s association with avoidance-related behaviors.

Under the broad hypothesis that the IBACS would detect levels of symptoms of complications in grieving, we formulated two sets of operational hypotheses. The first set of hypotheses concerned the IBACS’s discriminative ability. We hypothesized that, using Receiver Operating Characteristic (ROC) curve analysis, the IBACS would demonstrate the ability to differentiate complex grief from cases of normal grief. The second set of hypotheses, which addressed the concurrent and predictive validity of the IBACS, stated that the IBACS would demonstrate medium to strong correlations with related measures in comparative instruments, as well as similar clinical implications.

## Method

Because the present research was designed to test the validity of a new assessment instrument, details of this instrument are relevant to the participants and procedure sections of this study. For this reason, we will provide a description of the materials used in the analysis before presenting a description of study participants.

### Materials

The Indicator of Bereavement Adaptation—Cruse Scotland (IBACS). The instrument examined in the present study, the IBACS, was developed to assess the severity of grief symptoms and the risk of developing complications in grieving. The IBACS was designed by Cruse Bereavement Care Scotland (CBCS) to fulfill two main purposes: first, to provide a standard, consistent client intake process that could be conducted by professional counselors, volunteers, and staff across CBCS’s Scotland-wide service; and second, to support a stepped model of care by assessing the severity of bereaved clients’ grief symptoms and risk of complications. As such, the content needed to be bereavement specific and to assess grief symptoms as well as recognized risk factors for developing complications in grieving. In order to ensure the instrument’s acceptability, the research team vetted the instrument with CBCS counselors across Scotland, resulting in the following list of criteria for the instrument. It was agreed that items needed to be 1) brief, phrased in culturally acceptable terms and clear, accessible language 2) consistent across all service points; 3) easily scored, so that results of the assessment would be available directly at the conclusion of the assessment interview, and 4) designed to factor interviewers’ observations and insights regarding participants’ risk levels into the final assessment.

#### IBACS design

In order to meet the requirements detailed above, the IBACS needed to accommodate two somewhat conflicting needs: 1) the requirement for a consistent, structured assessment instrument, and 2) the need for an instrument that made allowances for a degree of observational (semi-structured) risk assessment and expert opinion. It was therefore decided to divide the IBACS into two sections: the risk factor assessment and the grief symptom self-report.

Part I: Risk factors. The risk factor assessment in the first section consists of a semistructured interview. Questions address the interviewee’s relationship with the person who died, the circumstances of that person’s death, as well as the interviewee’s previous experiences with grief, pattern of attachment, and other sources of stress or support. The semistructured design of this section of the interview allows a breadth of issues to be raised and discussed. In particular, the design enables participants to bring up pressing matters or to discuss their circumstances—issues that might otherwise be missed in the confines of a structured question set. The semistructured interview approach also permits the interviewer to conduct further inquiry where needed in order to document the magnitude of the risk of complications that the participant is experiencing. Specific guidelines are provided to calculate the number of risk factor points to allot based on the risk factors that emerge in the course of the interview. Risk points range from 0 to a maximum of 7.

Part II: Grief symptoms. The second component of the interview, the grief symptom self-report, consists of a structured questionnaire that measures a number of grief-related symptoms. The individual questionnaire items were primarily drawn and adapted (with permission) from the Inventory for Complicated Grief [16,30]. Selections were made by a panel of grief researchers and experienced bereavement support practitioners, and were chosen to provide a comprehensive but brief measure of loss-oriented behaviors (a mix of separation distress and traumatic distress items), along with two items (reverse-scored) measuring restoration-oriented behaviors, specifically personal growth. There are 12 items in this section of the interview, rated on a qualifying scale ranging from 0 (“Not at all”) to 4 (“All of the time”). Two items address suicidal ideation (“I have thought about ending my own life” and “I have done reckless things because I really don’t care what happens to me”). In the present study, responses to individual IBACS items were not available; however, internal consistency was assessed in a previous study [31]. In that study, with a sample of *N* = 331, internal consistency was found to be acceptable, with a Cronbach’s alpha of .75. The IBACS scoring process, cutoffs, and interviewer training process are discussed in the next section. The sum score of the IBACS, comprising the grief symptom section subtotal and the risk assessment score, can reach a maximum of 55 points.

To ensure the IBACS would be accessible to adults from a variety of educational backgrounds, a Flesch Reading Ease analysis was conducted using Microsoft Word 2011 (v14.4.9). Results indicated a readability score of 81.0 (out of 100), which can be interpreted as “Easy to read / conversational English”. A Flesch-Kinkaid Grade Level analysis was also conducted using the same program. The analysis indicated the text was accessible at the 4.5 grade level. At this grade level, the text would be comprehensible to people with a primary school level of education.

### Validated Measures

In the present study, four validated assessment measures were selected to provide a standard against which to measure the IBACS’s convergent and predictive validity. While all four instruments are widely used to assess symptoms of grief among bereaved people, two were designed to measure general psychological symptoms, and the other two measured symptoms for specific conditions prevalent among bereaved people: post-traumatic stress symptoms and grief-specific symptoms.

### General psychological symptoms

*1*. *Clinical Outcomes in Routine Evaluation* (*CORE)*. The CORE is widely used by private and NHS psychological support service providers across the UK to assess a variety of psychological symptoms. It was designed to be a brief, accessible instrument for use on a regular basis to measure symptom levels over time [32]. The CORE consists of 34 items that can be subdivided into four domains: symptoms (12 items), with item clusters around anxiety, depression, physical problems, and trauma; risk of harm (6 items), including clusters around risk to self and risk to others; social functioning (12 items), including clusters around general functioning, close relationships, and social relationships; and well-being (4 items). The CORE is assessed with a 5-point qualifying rating scale (from 0, “Not at all”; to 4, “Extremely”). Sample questions include: “I have felt like crying” (problems/symptoms), “I have been physically violent to others” (risk of harm), “I have felt OK about myself” (well-being), and “Talking to people has felt too much for me” (social functioning). The CORE has demonstrated very good test—retest reliability (.75–.95; [32]). Construct validity has been shown through convergence with conceptually similar instruments [32,33]. A high degree of scale reliability was also demonstrated in the present study (α = .90).

*2*. *The Symptom Checklist (Symptom Checklist 90 –Revised; SCL-90-R)*. The Symptom Checklist 90 -Revised consists of a 90-item self-report that can be used with both clinical and general populations [34,35]. Items are measured on a 5-point qualifying rating scale that ranges from 1 (“Not at all”) to 5 (“Extremely”). Responses to items are added together to create a total score that comprises the Global Severity Index. Items are further categorized into nine domains, which include anxiety, depression, hostility, interpersonal sensitivity (feelings of inadequacy), obsessive-compulsive, paranoid ideation, phobic anxiety (a persistent fear response with specific triggers, including agoraphobic symptoms), psychoticism, and somatization [36]. Construct validity has been established for all domains [36], while the depression, phobic anxiety, and interpersonal sensitivity subscales have also been subsequently validated as unidimensional scales [37]. Population norms and normative data are available for a variety of psychological conditions. Reliability has been demonstrated through appropriate internal consistency measures ranging from *α* = .74 to .97 [35]. In the present study, indications of scale reliability at baseline were extremely high (α = .98).

### Symptoms of Post-Traumatic Stress

*The Impact of Event Scale—Revised*. The Impact of Event Scale—Revised (IES-R; [38]) was designed to assess symptoms associated with post-traumatic stress. Symptoms are measured through 22 items for a total subjective stress assessment, and can be further divided into three subscales: intrusion, avoidance, and hyperarousal. The instrument is administered as a self-report that refers to a certain life event (in the present study, a bereavement). Individual items present a specific difficulty, and participants indicate to what extent that difficulty has distressed or bothered them over the last 7 days, using a qualifying rating scale. Sample questions include: “I felt watchful and on-guard” (hyperarousal), “I tried to remove it from my memory” (avoidance), and “Pictures about it popped into my mind” (intrusion). High test—retest reliability (*r* = .89 to .94) has been reported [38]. Convergent scale validity has also been demonstrated through a strong correlation (*r* = .84) with the PTSD Checklist [39]. Strong scale reliability was also demonstrated in the current study sample at baseline (α = .93).

### Grief-specific symptoms

*The Inventory of Complicated Grief*. The Inventory of Complicated Grief (ICG) is a bereavement-specific, 30-item instrument designed to assess the severity of symptoms associated with CG that the respondent has experienced over the previous month [30]. (For clarity we use the current name of the instrument, the ICG, in this manuscript, although the 30-item version published with the above citation was originally known as the Inventory of Traumatic Grief.) Different versions of the instrument are available under the name Inventory of Complicated Grief, including the most recent version, the Inventory of Complicated Grief-Revised (ICG-R). The ICG has demonstrated reliability through strong internal consistency (Cronbach’s α = .95; [30]). ICG items are measured using a 5-point frequency rating scale ranging from 1 (“Almost never”) to 5 (“Always”). Items form a single construct and address symptoms of separation distress and cognitive, emotional, and behavioral disruption [30,40]. Construct validity for the ICG was demonstrated through convergence with the outcome of a structured clinical interview for traumatic grief (the Traumatic Grief Evaluation of Response to Loss; [30]). It was further examined using the Dutch version of the instrument [41], with convergent validity shown through a high correlation (*r* = .71, *p* < .05) with a grief-specific inventory (The Texas Revised Inventory of Grief), and a moderate correlation (*r* = .59, *p* < .05) with a depression measure (the Beck Depression Inventory). In the present study, baseline analyses also demonstrated a high degree of reliability (α = .96). Although it is not recognized as a distinct diagnosis in *The Diagnostic and Statistical Manual of Mental Disorders* (5^th^ ed.; DSM–5 [42]; [43]), CG is formulated as a collection of bereavement-related symptoms causing extreme distress, which is viewed to be distinct from depression or anxiety [40,44]. Items include the statements: “I feel myself longing and yearning for [him/her],” “I see [him/her] stand before me,” and “I feel disbelief over [his/her] death.”

The aggregate score of the ICG is commonly used as an indication of symptom severity in grief-related research. Different methods have been developed for calculating *caseness*, a dichotomous cutoff score for CG. In the present study, we used a method created by Prigerson and Jacobs (2001) [30], which entails five criteria to establish caseness. In the present study, participants fulfilled three of these criteria at study enrollment (they were bereaved of a “significant other”; more than a minimum of two months had elapsed since the death; and they self-reported a severe degree of impairment stemming from their grief. The other two criteria stipulated a medium-to-high (self-report) rating on the separation distress symptom cluster (with at least three of five statements receiving a response of “4” or higher), and the traumatic distress symptom cluster (with at least six of the twelve items receiving a response of “4” or higher. (To accommodate clinical use, in regular practice a professional’s opinion of caseness may also be factored into the final score.)

### Participants

Recruitment for the study took place between January and December 2011 as part of a larger efficacy study of bereavement intervention, “Coping with Bereavement in Scotland.” Ethical review of the study was conducted by the NHS East of Scotland Research Ethics Committee 1 and approval was granted in September 2010. The sample of the larger study consisted of adult residents of Scotland (age 18+) who had been bereaved for at least six months, had requested bereavement support from CBCS, had yet to receive counseling services, and had no cognitive disabilities. Approximately 1,600 adults were approached to participate in the larger study, and received study recruitment packs containing information about the purpose of the study, the voluntary nature of participation, and how their confidentiality would be protected. Participants returned their signed consent forms via post. No compensation was offered for participation. Approximately 21% (*n* = 341) agreed to participate. Of these, 196 adults (44 male, 152 female) were assigned to the no-intervention control condition and were simultaneously enrolled in the present study. Assignment to conditions was quasi-randomized, working within the framework of the larger study’s naturalistic design. Participants who could not receive counseling support for logistical reasons (due to a waiting list in their local service areas, personal scheduling conflicts, or living too far away to reach a CBCS location) were assigned to the control condition.

Participation in the present study was limited to those people enrolled in the no-intervention control group of the larger study to avoid any confounding effects that intervention might have on follow-up questionnaire results. Table 1 presents the distribution of participants by age, gender, relationship to the deceased, and the deceased persons’ causes of death.

**Table 1:** Participant Demographics*.

		Male	Female	Total**	
		*n* = 44	*n* = 150	*N* = 194	
Participant Age					
	Mean	51.86	48.09	48.94	
	Standard Deviation	11.32	14.15	13.63	
Relationship to the Deceased					
*The deceased person was*:					
	Child	3	15	18	9%
	Parent	17	63	80	41%
	Partner	22	53	75	38%
	Sibling	1	12	13	7%
	Other relative / friend	1	7	8	4%
Cause of Death					
	Accident	3	10	13	7%
	Homicide	0	1	1	<1%
	Illness (Acute/chronic)	35	109	144	74%
	Suicide	4	10	14	7%
	Unknown	2	20	22	11%

*Data in the table are frequencies except where otherwise indicated.

** Percentages do not sum to 100 owing to rounding.

### Procedure

IBACS interviews (both Parts I and II) were conducted in sessions held either over the telephone or in person according to standard CBCS operating procedures by trained CBCS volunteers and staff. Participants also completed self-report postal questionnaires at intake (baseline, following the IBACS) and at follow-up after 18 months. IBACS scores were assessed by adding the risk of complication points (0 to 7) from Part I to the sum of the self-report responses from Part II (0 to 48 points) for a sum total score between 0 and 55 points. Preliminary cutoff scores for the assessment were provided as guidelines to indicate the level of added risk that the client is experiencing complications or will develop complications in grieving. These cutoff scores were experimental and were based on face validity as determined by a committee of bereavement research specialists and experienced bereavement support practitioners. As such, they also required validation. Participants whose IBACS response sums measured 18 points or lower were considered to be following a normal grief trajectory and not to be in need of bereavement support at that time. Intervention was recommended for participants with IBACS results of 19 and above.

The IBACS was designed to facilitate client allocation to categories of support within a stepped model of care. At CBCS, following an IBACS interview, a client would be allocated to one of three intervention categories, tiered according to the complexity of symptoms and degree of risk, or to a fourth *no intervention/watchful waiting* category. Table 2 presents guidelines for interpreting IBACS results for a stepped model of care.

**Table 2:** IBACS outcome guidelines for intervention.

IBACS Result	Recommended intervention
0–18	No intervention
19–28	Skilled listener intervention
29–38	Advanced skills listener
39–55	Counsellor

A different protocol was developed at CBCS for IBACS clients who reported high levels of suicidal ideation, indicated by a score of 6 points or higher on the two-question suicide subscale in Part II of the interview, or indicated verbally by the participant during Part I. Interviewers were trained to disregard the rest of the IBACS results in such circumstances and follow a suicide protocol, which included the involvement of outside specialized support depending on the severity of intent. Similarly, since substance abuse problems require attention before bereavement support can be provided, clients who indicated advanced substance abuse problems were to be referred to specialized substance abuse support resources as a precursor to grief intervention. Additional instructions were also provided for supporting study recruits with an IBACS result of 18 or lower who presented with elevated symptoms of distress that was not bereavement-specific. For example, if a participant presented with problems related to domestic violence, the interviewer would refer the participant to an appropriate support resource.

#### Training for the IBACS

A condensed training module for delivering the IBACS was developed for the provision of consistent, comprehensive instruction to CBCS volunteers and staff. No professional qualifications or counseling skills were required of trainees beyond basic interpersonal skills. Training consisted of an online learning module followed by two group training workshops led by experienced professional counselors who are also trainers approved by COSCA, Scotland’s professional body for counseling and psychotherapy. The workshops were spaced several weeks apart and included supervised, role-playing dyad assignments and a discussion of techniques and procedures for a productive interview. In between these workshops, trainees conducted three trial IBACS sessions with supervision provided by experienced professional practitioners.

In addition to preparation for conducting Part I of the IBACS, the interview component, the training process also included preparation for administering Part II of the IBACS, the structured symptom self-report. Interviewers were trained to encourage clients to complete the items, but to maintain neutrality with respect to the nature of the responses. For example, if a client remarked, “I’m not sure how to respond to this one,” training would instruct an interviewer to help the client refocus, gently reminding the client of the instructions, “just think about how you’ve been feeling over the last few weeks” or “take a moment to clear your thoughts, then read the item again… what’s the first response that comes to your mind?” Interviews for the present study were conducted by experienced IBACS interviewers who had completed the IBACS training module.

#### Participant Flow

At baseline, the sample numbered *N* = 196. Sixty-seven participants participated in the follow-up measure, which occurred 18 months later. A relatively high attrition rate was expected in the sample given the reports of previous longitudinal bereavement studies [45] and also due to the fact that participants in the present study constituted the no-intervention control group of a larger study, which may have made retention more challenging. In order to identify or rule out any potential attrition-led sources of bias in the sample, a logistic regression analysis was conducted to determine whether dropout at follow-up could be predicted by participants’ age, relationship to the deceased person, education level, time elapsed since the bereavement, severity of grief-related symptoms at baseline (as measured by the ICG), or the deceased person’s cause of death. The sample size for the regression analyses (*n* = 186) was slightly smaller than the total study sample because 10 cases were missing at least one variable. Results indicated that the full model, including all six independent predictor variables (dummy coded), was statistically significant (*x*^2^(7, *n* = 186) = 18.99, *p* < .001), suggesting that there is a pattern in attrition. The model as a whole explained 9.7% (Cox and Snell R square) to 13.3% (Nagelkerke R square) of the variation in dropout, and correctly identified 66.1% of cases. This was, however, only a minimal increase over the baseline measure of 64.5% of cases correctly identified. Only one variable was found to make a statistically significant contribution to the model: relationship to the deceased. Those participants bereaved of a parent were 3.3 times more likely to drop out at follow-up compared to those who were bereaved of a child (OR = 3.3, p < .001). The increase offered by the total model in predicting dropout was minimal, however, and it appears unlikely that this attrition pattern would affect results.

### Data Analysis and Detailed Hypotheses

Two steps were taken for the validation of the IBACS in the present research. Below we discuss the operational hypotheses for each step, and the techniques used to test them. Statistical significance for the present study was set at *p* < .05.

#### 1. Discriminative ability

Step one was to test the IBACS’s discriminative ability. At present, there is no gold standard for measuring grief-related symptoms and complications [8,46]. In the absence of such a standard, we selected a high level of severity of symptoms associated with the construct of CG to serve as the indicator that a participant would benefit from tertiary intervention. This level of severity was represented by a dichotomous variable for CG *caseness*, which was calculated using participants’ ICG responses at baseline following guidelines provided by Prigerson and colleagues (1995) [16]. To test the IBACS’s ability to discriminate between positive and negative caseness, we used ROC curve analysis. ROC curve analysis is widely used in diagnostic test assessment to evaluate the inherent validity of a test in terms of sensitivity and specificity of diagnosis [47]. In the present study, participant IBACS scores were entered as the test variable; the baseline caseness variable served as the dichotomous outcome variable. Then, to assess the validity of the IBACS as an indicator of caseness over time, the ROC curve analysis was repeated using the IBACS test variable at baseline. A second caseness outcome variable was calculated from ICG responses at follow-up.

Several guidelines for interpreting ROC curve results have been established for diagnostic tool assessment. Test performance is assessed by measuring the area that is calculated to lie under the curve (AUC; [48]). Guidelines in the literature to facilitate diagnostic tool assessment suggest that an AUC of .9 to 1.0 indicates high accuracy of test performance, .7 to .9 indicates moderate accuracy, .5 to .7 indicates lower accuracy, and below .5 indicates that the instrument performs no better than chance [49]. A grief-specific indication was also found in a study by Guldin and colleagues (2011) [50]. Using ROC curve analysis to compare a number of instruments’ abilities to detect CG (calculated using an ICG cutoff score of 43), the highest AUC produced was .83 by the Beck’s Depression Inventory.

Given the grief-specific focus of the IBACS and the influence of the ICG on its development, the IBACS was expected to successfully discriminate cases of CG. Specific operational hypotheses were formulated predicting that the ROC curve’s AUC—which assesses performance of the IBACS in detecting caseness—would be above .8, demonstrating moderate accuracy. A further hypothesis was developed for using ROC curve analysis to test the predictive ability of the IBACS in discriminating caseness over time. Given indications in the literature that grief symptom levels generally decrease over time [8], it was expected that the IBACS at baseline would continue to discriminate cases of CG with decreased but moderate accuracy, expecting an AUC of between .7 to .8. This analysis was conducted using IBACS scores at baseline as the predictor variable and a caseness variable at follow-up as the dichotomous outcome variable.

ROC curve analysis also facilitates the calculation of optimal cutoff thresholds for sensitivity and specificity. Due to the lack of reliable data concerning the prevalence of high levels of complications in grieving among a help-seeking bereaved population, it was not possible to inform our analysis with priors based on epidemiological prevalence. Instead, a maximal Youden’s *J* statistic was selected as a gold standard. The maximal Youden’s *J* (J = maxc {Se(c) + Sp(c) −1}) indicates the criterion value on the ROC curve when specificity and sensitivity are equally weighted and disease prevalence is set at 50% [51]. We hypothesized that the criterion associated with the maximal Youden’s *J* would correspond to the IBACS cutoff score criterion of 39, which indicates the most advanced intervention level, the *counselor* category. This category was designed to accommodate bereaved people for a tertiary intervention.

#### 2. Concurrent and predictive validity

Concurrent validity was assessed by running correlation analyses with four selected assessment instruments at baseline. To minimize the effect of participant attrition on results, listwise deletion was used for each scale to include only data from participants with complete datasets at both timepoints. As detailed above, the instruments included in the analysis were: the CORE, ICG-R, IES-R, and SCL-90-R. Following Cohen (1988) [52], we considered a correlation of 0.2 to be low, a correlation of 0.5 to be medium strength, and a correlation of 0.8 to be strong. A two-sided significance level (*α* = .05) was used in all analyses. At baseline, a positive correlation with a large effect size was expected with all four instruments and their subscales, all of which measured psychological symptoms that ranged from closely associated with bereavement (CG) to not being congruous with bereavement (psychoticism). Given the ICG’s grief specificity and influence on the IBACS’s development, it was hypothesized that the IBACS would demonstrate:

1. The strongest correlation with the ICG, with a medium-to-high degree of correlation, articulated for present research purposes as ranging from *r* = .60 to .70.
2. A medium-strength correlation with subscales assessing anxiety, depression, hyperarousal, and intrusive thoughts. For research purposes, the range of correlation was specified as *r* = .40 to .59.
3. Low degrees of correlation (between .30 and .39) with scales assessing more general psychological symptoms, including the sum scores of the CORE and SCL, measures of well-being, and other psychological symptoms which are less frequently associated with grief, such as psychoticism and paranoia.
4. Similarly low correlation with an avoidance subscale, again specified for research purposes as between .30 and .39.

Following this set of correlations, a second set of correlation analyses was calculated to test the predictive validity of the IBACS. In this step, correlation was assessed between the IBACS measure at baseline and the follow-up measure of the same four assessment instruments after 18 months had passed. Again given indications in the literature that symptom levels decrease over time, we expected to find that correlations would also decrease due to greater variation.

Finally, to consider the concurrency of the clinical implications of results across instruments, norms and cutoff scores for the two general symptom instruments, the CORE and the SCL-90-R, were compared to group means of IBACS intervention categories, which are based on IBACS cutoff scores. (It was decided to exclude the ICG and IES-R from this process because the analysis would be redundant for the ICG after the ROC curve analysis, and IES-R cutoff indications are specific to a distinct condition—post-traumatic stress disorder—which may be present in some bereaved participants.) It was hypothesized that the means of IBACS scores by intervention category would correspond to increasing mean scores on the CORE and SCL-90-R. It was also expected that the clinical recommendations yielded by the IBACS would indicate the same degree of case complexity as the other instruments, and to the degree indicated in the clinical interpretations of scores on the instruments to which it was compared.

## Results

### 1. Discriminative Ability

**Fig 1:**
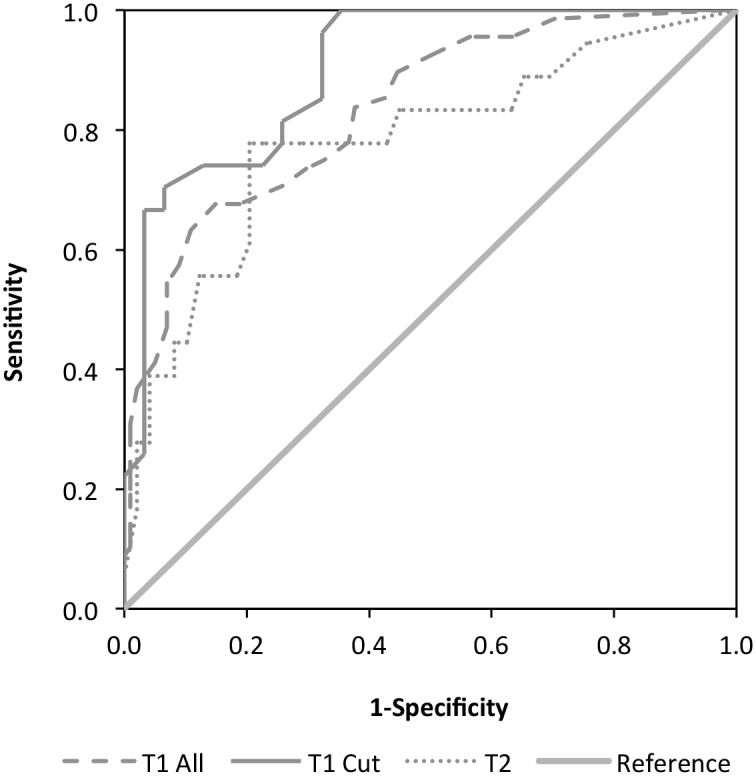
ROC Curve Analysis for CG Caseness at Time 1, Time 1 Cut (exclusively study completers) and Time 2.

Fig 1 illustrates the results of the ROC curve analysis at baseline. The sample for the ROC curve analysis at baseline (*n* = 169) was somewhat smaller than the total study sample size. This was due to the exclusion of 27 participants from the analysis because they had missing data on their ICG measures, which prevented the calculation of a caseness variable. The results of the analysis were statistically significant (*p* < .001) and indicated an AUC of .84, with a 95% confidence interval between .77 and .90. Interpreting the results using the indications provided by Greiner, Pfeiffer, and Smith (2000) [49] confirmed our hypothesis that the IBACS is a good diagnostic instrument for assessing CG.

The Youden’s *J* statistic was calculated (.05) with a sensitivity of 67.65 and a specificity of 85.15. The value was associated with a criterion of >32 on the IBACS. This criterion was lower than our hypothesis of 39, which corresponded with the practice-based cutoff indicating the intervention category with the highest degree of severity. Its placement is approximately halfway along the current IBACS intervention scale. The criterion of 32 corresponds with the *advanced skills listener* category of intervention. Thus, within the existing IBACS allocation system, the majority of participants in the advanced skills listener category, and nearly all participants in the counselor category, can be expected to be positive for caseness. Participants in this system’s skilled listener category fall under the >32 criterion, and would be expected to have negative caseness scores.

The second ROC curve analysis, using the IBACS at baseline and a caseness outcome variable calculated at follow-up (*n* = 67, *p* < .001) produced an AUC of .78 with a 95% confidence interval between .65 and .92. (N.B.: Study attrition resulted in a smaller follow-up sample. For comparison purposes, the ROC curve analysis at baseline was also run using a sample restricted to those participants who were present at both timepoints.) A DeLong’s test was conducted to compare the AUCs of the ROC curves that illustrated the performance of study completers at Time 1 and Time 2 [53]. The DeLong’s test indicated that there was no significant difference (*p* = .314) between the AUCs of the ROC curves at the two time points (D = 1.012, *df* = 86.86). These results indicate that the IBACS at baseline is a fair predictor of CG in participants 18 months later.

### 2. Concurrent and Predictive Validity

Table 3 shows the matrix of correlations between the IBACS and the instruments examined. As predicted, a positive correlation (*p* < .001, 2-tailed) was found between IBACS results at baseline, all four questionnaires (ICG, SCL-90, IES-R, and CORE-R) and their subscales at baseline, with the exception of the SCL-90 subscale for hostility and the IES-R subscale for avoidance. Consistent with the expectation that the IBACS would correlate most closely with a grief-specific instrument, the IBACS demonstrated the strongest correlation with the ICG sum score (*r* = .82), with a large effect size. Baseline correlations between the IBACS and measures for anxiety (*r* = .46), depression (*r* = .41), hyperarousal (*r* = .40)—all of which have been associated with bereavement-related grief in the literature—fell within the predicted low-medium range. The IBACS’s correlation with a measure for avoidance was even lower than predicted (*r* = .16), with a small effect size. A surprising, medium-strength correlation was found between the IBACS and measures for psychoticism (*r* = .54) and paranoid ideation (*r* = .49), as well as obsessive compulsive symptoms (*r* = .44).

**Table 3:** Correlation between the IBACS and comparator instruments at baseline and follow-up.

	Baseline	Follow-up	n*
Symptom Checklist 90-Revised (SCL-90-R)			
SCL-90-R global severity index	.48**	0.61**	61
SCL-90-R somatization	0.29*	0.52**	55
SCL-90-R obsessive compulsive	0.44**	0.66**	59
SCL-90-R interpersonal sensitivity	0.38**	0.52**	60
SCL-90-R depression	0.41**	0.58**	51
SCL-90-R anxiety	0.46**	0.63**	54
SCL-90-R hostility	0.22	0.40**	62
SCL-90-R phobic anxiety	0.44**	0.52**	60
SCL-90-R paranoid ideation	0.49**	0.58**	60
SCL-90-R psychoticism	0.54**	0.62**	57
Inventory of Complicated Grief-Revised	.82**	.71**	55
Impact of Event Scale- Revised (IES-R)			
IES-R sum	.34**	.57**	65
IES-R avoidance	0.16	.43**	65
IES-R hyper-arousal	.40**	.59**	65
IES-R intrusive thoughts	.35**	.56**	65
Clinical Outcomes in Routine Evaluation-Revised (CORE)			
CORE Sum	.71**	.68**	50
CORE functioning	.35**	.40**	58
CORE problems	.60**	.56**	57
CORE risk	.64**	.56**	61
CORE well-being	.32**	.27*	63

*Sample sizes vary due to listwise deletion

**p < .01

Scores on two general symptom assessment instruments, the CORE and SCL-90, were used to provide justification for the appropriateness of the IBACS cutoff scores. In order to do this, separate one-way analyses of variance (ANOVAs) with a Bonferroni correction were conducted to compare the IBACS cutoff points with the scores and severity levels of the CORE and the SCL-90-R at baseline. Participants were divided into the four intervention categories indicated by their IBACS scores (as discussed above): *no intervention* (0–18), *skilled listener* (19–28), *advanced skills listener* (29–38), and *counselor* (39 to 54).

Table 4 lists the IBACS intervention categories and their corresponding CORE and SCL-90 cutoffs. Due to missing (incomplete) data, some cases were excluded from the analysis. Each IBACS intervention category corresponded with an increasingly advanced degree of case severity, which all fell above the clinical threshold. There was a statistically significant difference in CORE scores for the four categories; *F* (2,179) = 42.56, *p* < .001. The effect size, calculated using eta squared, was medium (.32). ANOVAs comparing IBACS categories to SCL-90-R performance were conducted using separate global norms established for women and men [54]. The results for both analyses were significant at the *p* < .001 level with a Bonferroni adjustment; for women, the results indicated *F*(3, 145) = 16.46; and for men, *F*(3, 42) = 8.71. In sum, participants in different IBACS intervention categories also had distinct results on the CORE and the SCL-90-R, as demonstrated by the statistically significant differences between the IBACS intervention groups that were found in the results of these two assessment instruments. The mean CORE score and SCL-90-GSI mean for each IBACS intervention category (listed in Table 4) demonstrated that these categories correspond with increasing symptom complexity. A comparison was also made between the case severity level indicated by the IBACS and the cutoffs and clinical implications associated with each IBACS category mean on the CORE and the SCL-90-R. This comparison revealed that IBACS categories obtained mean scores on the CORE and SCL-90-R that corresponded with increasing degrees of case severity above the clinical threshold.

**Table 4:** Cut Off Score Convergence at baseline.

		IBACS Intervention Categories		
		Skilled Listener	Advanced Skills Listener	Counsellor
IBACS				
	*N* = 186* (*excluding 8 “no intervention”)	*n* = 90	*n* = 61	*n* = 35
	Mean	22.90	32.89	43.29
	Standard Deviation	4.56	4.22	6.38
	Score Range	18 through 28	29 through 38	39 through 55
Clinical Outcomes in Routine Evaluation—Outcome Measure (CORE-OM)				
	*n* = 182	*n* = 89	*n* = 59	*n* = 34
	Mean	50.21	65.76	93.35
	Standard Deviation	22.77	23.76	23.97
	Clinical Indication	Mild severity (Clinical Range)	Moderate severity (Clinical Range)	Severe (Clinical Range)
Inventory of Complicated Grief Revised (ICG-R)				
	*n* = 164	*n* = 80	*n* = 55	*n* = 29
	Mean	83.97	103.00	131.00
	Standard Deviation	22.12	20.12	12.96
	Positive “Caseness”	19.8% (*N* = 16)	48.1% (*N* = 26)	92.6% (*N* = 25)
Symptom Checklist-90-Revised (SCL-90-R) Global Severity Index				
	n = 184	*n = 90*	*n = 59*	*n = 35*
	43 Men, 149 Women	21 Men, 69 Women	13 Men, 46 Women	6 Men, 29 Women
Men	Mean	1.00	1.69	2.45
	Standard Deviation	.63	.73	1.01
	Clinical Indication	Low	High	Very High
Women	Mean	1.47	1.85	2.55
	Standard Deviation	.82	.73	.86
	Clinical Indication	Above Average	High	Very High

As shown in Table 4, the mean scores in the three IBACS intervention categories corresponded with CORE results that were above the clinical threshold in the CORE classification system. They followed a similar pattern of increasing severity, with the IBACS skilled listener category (*M* = 50.21, *SD* = 22.78) indicating (clinical) mild level severity on the CORE, the IBACS advanced skills listener category’s CORE mean (*M* = 65.76, *SD* = 23.76) indicating (clinical) moderate severity, and the IBACS counselor category’s CORE mean (*M* = 93.35, *SD* = 23.97) corresponding with the CORE’s (clinical) severe category.

Predictive validity was assessed by calculating correlations between the IBACS at baseline and the four instruments at follow-up after 18 months had passed. The relationship between IBACS results at baseline and the ICG at follow-up remained strong (*r* = .71), implying a medium-to-large effect size. Some change was demonstrated in the results from other instruments. The strength of the correlation increased between the IBACS at baseline and all IES-R measures taken at follow-up. While hyperarousal (*r* = .59) and intrusive thoughts (*r* = .56) demonstrated a medium-strength correlations, correlation with the avoidance subscale, which was very low at baseline, increased at follow-up to a low-to-medium-strength (*r* = .43) and was significant at the p < .001 level. Correlations between the IBACS and all the SCL-90 subscales were also stronger at follow-up. This included an increase in the unexpected medium-strength correlation found with baseline measures for psychoticism (increased to *r* = .62 at follow-up) and paranoid ideation, (*r* = .58 at follow-up). The correlation with the obsessive-compulsive symptom subscale was the strongest of the SCL-90 subscales at follow-up (*r* = .66), followed by anxiety (*r* = .63). Correlations with the CORE and its subscales declined slightly but remained consistently medium strength for the sum score (r = .57), as well as the problems (*r* = .56) and risk (*r* = .56) subscales. A weaker correlation with the CORE well-being subscale remained low (*r* = .27), while correlation with the functioning subscale increased slightly (*r* = .40).

## Discussion

The goals of this project were threefold: (a) to test the validity of the IBACS—an instrument developed for use by nonclinicians as well as professionals—against other valid measures, (b) to test the sensitivity of the cutoff point for intervention, and (c) to test the IBACS’s discriminative ability concerning complex grief reactions as demonstrated in discerning CG caseness over time. The IBACS was an effective instrument for detecting moderate to severe difficulties coping with grief. The ROC curve analysis showed that the IBACS performed with satisfactory sensitivity and specificity in indicating caseness of the CG construct at a given time. The IBACS also demonstrated concurrency with other valid assessment instruments as an effective measure of grief-related symptoms. Assessing the IBACS’s predictive validity, ROC analyses indicated only fair sensitivity to caseness over time. In contrast, the strength of correlation between the IBACS and the ICG, from which the caseness variable was calculated, remained equal over time.

The first set of hypotheses tested the strength of the relationship between the IBACS and a number of grief-related symptoms. A medium-strength relationship between the IBACS and subscales for anxiety, depression, and hyperarousal was confirmed. As expected, a weaker relationship between the IBACS and the IES-R avoidance subscale was also supported, given prior research indicating that overtly avoidant behaviors are low among bereaved people with symptoms of CG [28]. Instead, it has been theorized that in such cases, ruminative thoughts may themselves be a mechanism of avoidance [28].

One finding was the stronger than expected correlation between the IBACS and three subscales on the SCL-90-R: measures of obsessive-compulsive behavior, paranoid ideation, and psychoticism. Since prior research had not found a relationship between these constructs and bereavement-related grief symptoms, we examined the individual items in each subscale for possible explanations. We considered the standard instructions that participants had been given to respond to items based on how they had felt over the previous month. Additionally, the instruments were administered during a study on coping with bereavement, which included several grief-specific instruments and questions about bereavement-related circumstances. Reviewing the paranoia, psychoticism, and obsessive-compulsive subscale items with this in mind, it became apparent that non-grief-specific items might have been interpreted in terms of bereavement-related grief behaviors. For example, the obsessive-compulsive scale items described behaviors that included obsessive thoughts, which in a bereavement context could relate to intrusive thoughts about the deceased person and other reminders connected to bereavement. Checking behaviors and insecurities about task completion could also relate to specific difficulties in adjustment common to bereaved people. Items on the paranoia subscale, such as discomfort in public and feelings of being observed or standing out in a crowd, were also found to be relevant to post-bereavement adjustment. Lastly, the psychoticism subscale included items that addressed auditory and visual hallucinations. While hallucinations themselves are not generally considered symptoms of grief, it is not uncommon for bereaved people to report seeing or hearing the deceased person. Examining the correlation between the IBACS and these three distinct (and at first, seemingly unrelated) constructs was important for a number of reasons. It was a reminder of the need for bereavement-specific norms in general symptom questionnaires, and the importance of not assuming that general symptom questionnaires generate general (and not grief-related) responses in a bereaved population.

The second set of hypotheses concerning the IBACS’s ability to discriminate CG caseness was also confirmed using ROC curve analysis. The optimal cutoff point for the IBACS, which is >32, was established using Youden’s *J* statistic. We had expected to find parallels between the cutoff criterion for caseness and the most complex category in the current IBACS system, the counselor category. Instead, using a standardized prevalence rate and giving equal importance to sensitivity and specificity resulted in a criterion that closely mirrored the advanced listener category cutoff. This therefore suggests that not only the counselor category participants, but also those people in the advanced skills listener group present strong grief symptoms, whereas those in the skilled listener group are at risk of developing grief symptoms. Further evaluation of IBACS cutoff criteria is needed, preferably with a prevalence statistic specific to the help-seeking bereaved population. Moreover, while Youden’s *J* gives equal value to sensitivity and specificity, in practice, the necessary choice between caseness sensitivity and specificity should be carefully weighed. On the one hand, a false negative for CG could lead to a lack of intervention or assignment to a practitioner not suitably skilled to support the client. On the other hand, a false positive could mean pathologizing aspects of the normal human condition, such as grief, which could be even more detrimental.

Overall, the results support the validity of the IBACS as an intake assessment instrument for bereavement-related grief. While at present there is no gold standard against which to compare the assessment of bereavement-related grief symptoms and risk of complications (attributable in part to the fact that an extreme grief response, although universally recognized as suffering, is not currently a unique diagnosis [43]), the IBACS showed moderate concurrent validity with four widely used, validated assessment instruments, one of which was specific to grief responses. This represents an important step toward creating an effective intake instrument for nonclinicians. Such an instrument is of particular importance when considering current circumstances in bereavement care, where an estimated 70% of bereavement intervention services in the UK are provided by the nonprofit sector [22], and the large majority of bereavement support offered in Australia, Canada, Japan, the UK, and the USA does not entail targeted support or the use of a formal risk assessment at intake [55].

We have assessed the instrument in the delivery context for which the IBACS was designed: assessment in a nonclinical setting. In this context, the IBACS appears to perform satisfactorily. Two things need to be considered when looking at this outcome. First, the correlation analyses were conducted with the other assessment instruments exclusively using their rating scale symptom assessments. Second, if the other assessment instruments had actually been administered in a clinical setting, professional opinion would have contributed to the final assessment, and assessment outcomes may have been different. Furthermore, as with any instrument, additional analyses must be conducted to establish a comprehensive evaluation of its validity. One critical issue concerns the application of the IBACS in other (cultural) settings. The current study was conducted in Scotland, where the instrument was developed, including guidelines for risk factor assessment that were found to be culturally appropriate for a British population. It will be important to revisit these guidelines when using the instrument in other cultures where different social norms for expressions of grief may need to be considered. Similarly, it is important to re-evaluate optimal cutoff points to ensure that appropriate thresholds for intervention are in place in distinct cultural settings.

There were several limitations to our study. First, the naturalistic design prevented further steps for examining the efficacy of the IBACS as an assessment instrument. An item-level factor analysis was not possible given the lack of item-level data. Also, the decision not to disturb standard intake procedures precluded testing inter-rater reliability. This would have required duplicate interviews or the presence of multiple interviewers at each IBACS session (or via video) to compare risk point allocation. Another significant limitation was the lack of low-level responders in the dataset. Because the participant sample consisted of help-seeking bereaved people who were participating in a larger research study, those who scored under 18 on the IBACS were excluded from the dataset. To address this issue, additional research is recommended to conduct a psychometric validation using item-level responses and working with a sample that presents a broader spectrum of case complexity.

Finally, as for all longitudinal studies, participant attrition must be considered. Participant dropout reduced power in the correlation analyses at follow-up. Although the cause of study dropout is usually unknown, there are two issues worth considering. First, study design may have increased the likelihood of participant dropout; longitudinal studies using postal questionnaires have higher attrition rates than studies employing other methods, such as face-to-face interviews. Second, changing life circumstances for participants may have also impeded data collection due to a lack of accurate contact information. It is worth noting that low response rates are not uncommon in quantitative research among bereaved people [1].

Despite the limitations, the IBACS appears to be a good intake assessment instrument for bereavement intervention. Although a definitive assessment of its accuracy cannot be made at this time, the IBACS offers moderately concurrent results with instruments that were (atypically) administered without a clinical assessment. One advantage is that the IBACS is easy to use in nonclinical settings. It can be delivered by interviewers who have completed a basic training module. This is of particular importance in countries like the UK, as noted above, where the large majority of bereavement support is provided by non- or paraprofessional resources who lack the training and professional qualification to offer clinical interviews.

Consistent and informed assessment of grief symptoms and risk of complications is almost never offered; yet the scientific literature indicates the importance of assessment in creating positive outcomes for help-seeking bereaved people and for promoting the effective use of resources. An accessible instrument like the IBACS can fill a critical gap by enabling nonclinicians to assess bereaved people’s symptom and risk levels in order to determine whether they would benefit from bereavement intervention. This supports expanding the availability of bereavement assessment, allowing for more targeted interventions and fewer wasted resources.
